# Effect of *Ambrosia arborescens* Mill. ethanolic extract on breast cancer induced in rats

**DOI:** 10.14202/vetworld.2024.700-704

**Published:** 2024-03-25

**Authors:** Carmen R. Silva-Correa, Víctor E. Villarreal-La Torre, Gladys E. Lozano-Ciudad, Ricardo M. Gómez-Arce, Julio A. Castañeda-Carranza, Deivy Y. Dionicio-Rosado, María E. Cotrina-León, William A. Sagástegui-Guarniz, César D. Gamarra-Sánchez, José L. Cruzado-Razco

**Affiliations:** 1 Departamento de Farmacología, Facultad de Farmacia y Bioquímica, Universidad Nacional de Trujillo, Perú; 2 Departamento de Estadística, Facultad de Ciencias Físicas y Matemáticas, Universidad Nacional de Trujillo, Perú; 3 Departamento de Matemáticas, Facultad de Ciencias Físicas y Matemáticas, Universidad Nacional de Trujillo, Perú

**Keywords:** 7,12-dimethylbenz[a]anthracene, *Ambrosia arborescens*, breast carcinoma *in situ*, cancer, rat

## Abstract

**Background and Aims:**

*Ambrosia arborescens* Mill. (*A. arborescens*) is an aromatic plant used in traditional medicine as an anti-inflammatory, anti-tussive, anti-rheumatic, and anti-diarrheal agent. This study aimed to evaluate the effect of *A. arborescens* Mill. on a *Rattus norvegicus* var. *albinus*-induced breast cancer model.

**Materials and Methods:**

We collected *A. arborescens* from the province of Julcán, La Libertad Region, Per, and prepared an ethanolic extract using pulverized leaves macerated in 96° ethanol for 72 h with magnetic stirring. In the evaluation of anticancer activity, four experimental groups with 10 female rats each were formed: Group I (Control-7,12-dimethylbenz[a]anthracene [DMBA]), which received DMBA (single dose) and physiological saline solution for 4 months, and Groups II, III, and IV, which received DMBA (single dose) and 200, 400, and 600 mg/kg/day of the ethanolic extract of *A. arborescens*, respectively, for 4 months.

**Results:**

The DMBA control group presented histological characteristics of ductal carcinoma *in situ* with necrotic and inflammatory areas, whereas the *A. arborescens* extract group showed a decrease in tumor volume and recovery of the ductal duct.

**Conclusion:**

Ethanol extract of *A. arborescens* leaves decreases tumor development in rats with induced breast cancer, and this effect is dose-dependent.

## Introduction

Cancer is one of the main public health problems [1]. In 2020, almost 10 million deaths have been attributed to this disease, that is, one in six of those registered. Lung, liver, colorectal, gastric, and breast cancers are the main types of cancer that cause the highest number of deaths. In Peru, more than 69 thousand new cases of cancer are diagnosed each year, and it is estimated that more than 34,000 people die due to cancer. The most frequent cancers are prostate, breast, stomach, colon, and cervical [2, 3]. The pathophysiology of cancer explains the uncontrolled growth and spread of abnormal cells caused by an alteration of the regulatory mechanisms of cell growth, proliferation, and death, in addition to the fact that cancer cells are characterized by evading apoptosis or programed cell death, managing to invade various tissues and organs complicating the pathological process of this disease [4, 5]. Breast cancer is the most common type of carcinoma in women and the second most common cause of cancer-related mortality in women [6]. Invasive lobular carcinoma is the second most common histological subtype of breast cancer after invasive ductal carcinoma [7, 8].

According to the World Health Organization, the histopathological classification of breast carcinomas includes *in situ* carcinomas, invasive breast carcinomas of no special type, lobular, cribriform, tubular, mucinous, papillary, metaplastic carcinomas, carcinomas with medullary pattern, and carcinomas with apocrine differentiation [9]. Four molecular subtypes have been identified in breast cancer by applying gene expression profiles: Luminal A, luminal B, human epidermal growth factor receptor 2, and basal type [10]. To replicate the carcinogenic process, animal models have been used to chemically induce breast cancer in rats with 7,12-dimethylbenz[a]anthracene (DMBA) [11], a synthetic polycyclic aromatic hydrocarbon (PAH) [12], which is an environmental carcinogen.

The diversity of bioactive compounds in plants explains the discovery of anticancer agents from medicinal plants, which serve as prototypes for discovering and developing new medications [13]. In Per, one of the most representative and numerous families is *Asteraceae*, in which the species *Ambrosia arborescens* Mill. (*A. arborescens*), commonly called “Marco” or “Altamisa,” is found, an aromatic plant used in traditional medicine as an anti-inflammatory, antitussive, antirheumatic, and antidiarrheal agent [14, 15]. Phytochemical studies in the leaves of *A. arborescens* reveal the presence of eudesmane-type sesquiterpenes, diterpenes, and sesquiterpene lactones (damsin and coronopilin) that show antiproliferative and cytotoxic activity in breast cancer cell lines [16–18].

On the basis of these findings, this study aimed to evaluate the effect of the ethanolic extract of the leaves of *A. arborescens* on a breast cancer model induced in *Rattus norvegicus* var. *albinus*. This will allow the exploration of the pharmacological potential of this plant resource and support and strengthen existing studies, which will allow the development of new drugs in the future.

## Materials and Methods

### Ethical approval

This study was approved by the Ethics Committee of the Faculty of Pharmacy and Biochemistry of the Universidad Nacional de Trujillo (Approval Certificate No.: 011–2022/C.FAC.FARM).

### Study period and location

This study was conducted from April 2022 to December 2022. All procedures were performed in the Toxicology Laboratory, School of Pharmacy and Biochemistry, Universidad Nacional de Trujillo.

### Botanical material

*A. arborescens* leaves collected from Julcán Province, La Libertad Region, Peru. Specimens were taken to the *Herbarium Truxillense* (HUT) of the Universidad Nacional de Trujillo for taxonomic identification (HUT Code: 59575).

### Biological material

Forty specimens of *R. norvegicus* var. *albinus* females (average weight, 180–200 g; age, 2 months) selected at random were conditioned in the Animal Laboratory of the School of Pharmacy and Biochemistry of the Universidad Nacional de Trujillo and fed a standard diet and adjuvant water *ad libitum*.

### Preparation of the ethanol extract

Ethanolic extracts of *A. arborescens* leaves were prepared using pulverized leaves and allowed to macerate in 96° ethanol for 72 h with magnetic stirring. The extract was then filtered and placed in an oven at 45°C for 72 h to obtain the dry extract, which was stored at –20°C.

### Breast cancer induction [19, 20]

A single dose of 20 mg of DMBA (diluted in 1 mL olive oil) was subcutaneously administered and directly applied to the mammary gland area in rats. Four experimental groups were formed with ten female rats each: Group I (Control-DMBA), which received DMBA (single dose) and physiological saline solution for 4 months, and Groups II, III, and IV, which received DMBA (single dose) and 200, 400, and 600 mg/kg/day of the ethanolic extract of *A. arborescens*, respectively, for 4 months.

### Evaluation of anticancer activity [21]

Weekly evaluation of each of the rats was carried out to detect the presence of tumors through palpation in the area of the thoracoabdominal wall and the inguinal region, recording the time of appearance of the mammary tumors (latency); then, from this, the animals were euthanized with sodium pentobarbital 100 mg/kg intraperitoneal route and the tumors were extracted, which were measured using a Vernier, noting the height, length, and width of the tumor to determine the accumulated tumor volume using the following formula:

V = 1/2 [4/3π.w.l.h]

where V = accumulated tumor volume, w = width, l = length, and h = height.

### Histopathological analysis of tumors [22, 23]

The extracted tumors were preserved in 10% formalin solution for histopathological analysis, and slides stained with hematoxylin and eosin were obtained and observed using an optical microscope (Motic, Spain). In the histological analysis, necrosis and infiltration were observed.

### Statistical analysis

Evaluation of the results obtained in terms of tumor latency and tumor volume parameters was performed using the SPSS version 25 program for Windows^®^ (IBM Corp., NY, USA), with a statistical significance of p < 0.05.

## Results

### Evaluation of anticancer activity

**Figure-1:**
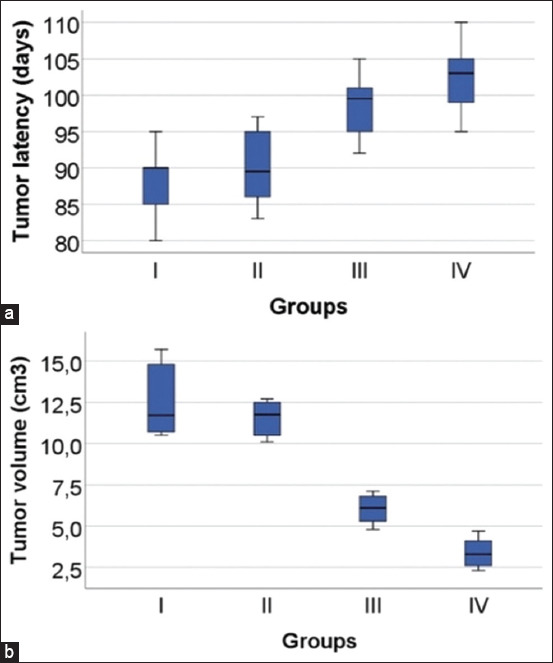
Effect of *Ambrosia arborescens* Mill. on (a) tumor latency (b) and tumor volume in rats with 7,12-dimethylbenz[a]anthracene (DMBA)-induced breast cancer. All data are presented as mean ± standard deviation, n = 10 per group, p < 0.05, Analysis of Variance, *post hoc* Tukey Honestly Significant Difference test.

Figure-1 shows the parameters recorded for the anticancer activity evaluation. Compared with the control group, a higher latency in days was observed regarding the appearance of tumor development and a decrease in tumor volume in the groups that received treatment with the extract of *A. arborescens* (p < 0.05). Group IV showed this significant difference.

### Histopathological analysis of tumor specimens

**Figure-2:**
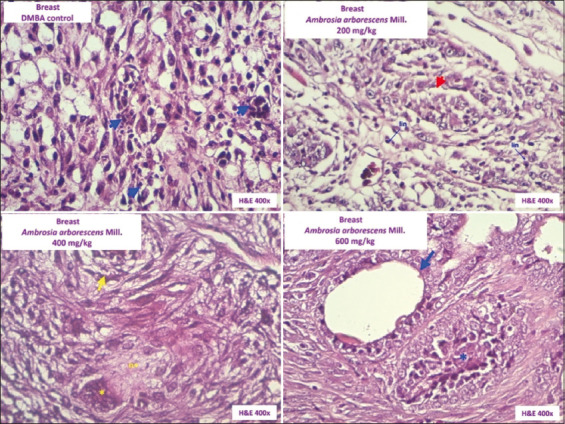
Photomicrographs of histological changes in breast tissue (hematoxylin and eosin, 400×).

Figure-2 shows the histological changes in breast tissue in the experimental groups. Histological characteristics corresponding to ductal carcinoma *in situ* with microcalcifications and evident necrosis in Group I (DMBA Control) are shown (A). Group II (*A. arborescens*-200) showed ductal carcinoma *in situ* with areas of necrosis and inflammation (B), and Group III (*A. arborescens*-400) showed ductal carcinoma *in situ* with areas of necrosis and intravascular lymphatic infiltration (B). However, insufficient recovery of the ductal epithelium (C) was observed compared with that observed in Group IV (*A. arborescens*-600), with characteristics of *in situ* ductal carcinoma but with evident recovery of the ductal epithelium.

## Discussion

PAHs have been identified as potent carcinogens and endocrine disruptors that are widely distributed in the environment and have been associated with the development of tumors in humans and experimental animals, including DMBA, which is used as the principal model for the study of mammary carcinogenesis in female rats [24]. The mechanism of DMBA is based on the metabolic activation of the compound in the mammary gland through the formation of the active metabolite DMBA-3,4-diol-1,2-epoxide, which is responsible for increased oxidative stress by interacting with cells and generating adducts with DNA [25]. These mechanisms cause lesions and hyperplasia of epithelial cells in the terminal ducts of the mammary gland [26].

Mammary tumor formation is evident 13 weeks after the initiation of DMBA administration [27]. As in the present study, subcutaneous administration of a single dose of DMBA (20 mg) in the mammary glands of rats resulted in breast tumors appearing in 88–102 days (latency), with a decrease in tumor volume, which was more evident in the group that received the ethanolic extract of the leaves of *A. arborescens* at a dose of 600 mg/kg (Figure-1).

Histopathological analysis in the control group that received the carcinogen DMBA showed a histological pattern of ductal carcinoma characterized by malignancy criteria [28]. According to the American Society of Cancer, this histological change (ductal cancers) corresponds to the most frequent type of breast cancer in women [29]. The histopathological results showed that specimens that received the ethanolic extract of the leaves of *A. arborescens* at the highest dose of 600 mg/kg showed a lower degree of histological severity in the characteristics of the tumor, with a lower number of inflammatory and necrotic areas, compared with the control group that only received DMBA, which presented a solid ductal carcinoma. In addition, administration of the extract resulted in histopathological changes such as recovery of the ductal epithelium in the mammary gland. These results suggest that the *A. arborescens* extract has antitumor activity in the development of cancer cells, but this effect depends on the dose of the extract.

The main compounds reported in phytochemical studies in the leaves of *A. arborescens* indicate the presence of eudesmans-type sesquiterpenes, diterpenes, and pseudoguaianolide sesquiterpenes such as damsin and coronopilin [30]. Pseudoguaianolides are sesquiterpene lactones having a cis- or trans-anellated lactone ring fuzed with seven five-membered rings [31], which have inhibitory effects on the transition of cells from the S phase to the M phase, blocking cell proliferation, which is related to their anticancer activity. Furthermore, damsin and coronopilin have inhibitory effects on both nuclear factor-kappa B (NF-κB) and signal transducer and activator of transcription 3 (STAT3) [32], indicating that they could be candidates for the development of powerful anticancer drugs because STAT3 and NF-κB cooperatively regulate several proinflammatory genes, such as interleukin-6, interleukin-11, chemokines, growth factors, and cyclooxygenase-2, all of which are crucial for maintaining a procarcinogenic inflammatory environment [33].

## Conclusion

The ethanol extract of *A. arborescens* Mill. decreased the latency and volume of tumors generated in rats with breast cancer induced by DMBA. This effect was more evident at the dose of 600 mg/kg, showing recovery of the ductal epithelium and a decrease in necrotic areas in the histopathological analysis, considering a dose-dependent effect.

## Authors’ Contributions

CRSC and VEVT: Collected the plant species and drafted the manuscript. GELC and WASG: Carried out the preparation of extract. CDGS, JLCR, and MECL: Maintained the animals during the investigation and administered the treatments. CRSC: Histopathological analysis. RMGA, JACC, and DYDR: Statistical analysis and image preparation. All authors have read, reviewed, and approved the final version of the manuscript.
